# Whole genome sequence data of *Stenotrophomonas maltophilia* SCAID WND1-2022 (370)

**DOI:** 10.1016/j.dib.2022.108694

**Published:** 2022-10-26

**Authors:** Ilya Korotetskiy, Ardak Jumagaziyeva, Bahkytzhan Kerimzhanova, Oleg Reva, Tatyana Kuznetsova, Sergey Shilov, Ludmila Ivanova, Natalya Zubenko, Raikhan Parenova, Zhanar Iskakbayeva, Bolatbek Baimakhanov, Aimana Bekmuhamedova

**Affiliations:** a JSC Scientific Center for Anti-Infectious Drugs, Almaty, Kazakhstan; b Centre for Bioinformatics and Computational Biology, Department of Biochemistry, Genetics, and Microbiology, University of Pretoria, Pretoria, South Africa; c JSC National Research Center of Surgery named after Syzganov, Department of Vascular Surgery, Almaty, Kazakhstan

**Keywords:** Hospital-associated pathogen, Antimicrobial resistance, Multidrug-resistant, Sequencing, Methylation

## Abstract

The whole genome sequence of a hospital infection agent, *Stenotrophomonas maltophilia* SCAID WND1-2022 (370), is reported. Raw PacBio generated reads and the genome sequence were deposited at NCBI under BioProject PRJNA754843. The genome comprises two replicons: 4,880,425 bp long chromosome comprising 4524 proteins and functional RNA coding genes and 38,606 bp long plasmid containing 40 CDS. Both replicons were methylated at third cytosine residues of AC*C*TC motifs. The taxonomic provenance of SCAID WND1-2022 (370) was determined by calculating sequence similarity to the reference genomes at NCBI that showed the highest 97.35% identity to *S. maltophilia* ISMMS4. Many antibiotic resistance and virulence genes were identified on the chromosome of *S. maltophilia* SCAID WND1-2022 (370), which include multiple efflux pumps, beta-lactamases, and genes involved in biofilm formation. The plasmid sequence was dissimilar to any known plasmid and seemingly was acquired from a distant microorganism. Plasmid-born genes possibly contributed to the virulence of the pathogens, but not to its drug resistance.

## Graphica abstract

**Figure:**
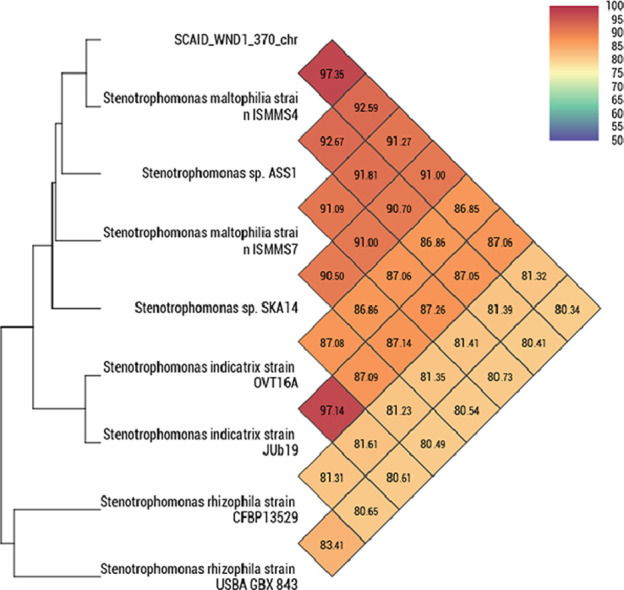

**Specifications Table**

**Table:** 

Subject	Microbiology
Specific subject area	Microbial genomics
Type of data	Raw reads and analysed genome of *Stenotrophomonas maltophilia* SCAID WND1-2022 *(370)*
How the data were acquired	Collection: The sample was obtained from the intensive care unit. The sample was cultured on selective and differential media. A wide spectrum of biochemical tests was used for identification. Next generation sequencing PacBio Sequel platform was used to generate long single-end reads. Software: DNA reads generated by PacBio were quality controlled using LongQC The whole genome sequence was assembled using Canu v2.0. Methylated nucleotide and genome methylation motifs were identified by software tools ipdSummary and motifMaker of the package smrtlink_10.1.0.119588. Genome sequences annotated using GenBank annotation robot PGAP and deposited at NCBI.
Data format	Raw data: PacBio BAM base calls with interpulse duration (IPD)Analyzed data: • FASTQ – DNA reads for genome assembly; • GBK – annotated whole genome sequences; • GFF – methylation predictions;
Description of data collection	DNA extraction was performed using the PureLink Genomic DNA Mini Kit (Invitrogen, USA). DNA was quantified by NanoDrop for purity. The DNA library was prepared using PacBio SMRTbell Express Template Prep Kit 2.0. Sequencing was performed using the PacBio Sequel-I (Pacific Biosciences) sequencing platform by Macrogen (Seoul, Korea).
Data source location	*•* Institution: Scientific Center for Anti-Infectious Drugs • City/Region: Almaty • Country: Kazakhstan • Latitude and longitude: 43°26′ N and 76°93′ E
Data accessibility	Repository name: GenBank: Data identification numbers: BioProject Accession Number: PRJNA754843, NCBI SRA Accession Number: SRR21079300, NCBI GenBank Accession Number: CP102942-CP102943 The direct URL to the data: (https://www.ncbi.nlm.nih.gov/bioproject/?term=PRJNA754843), (https://www.ncbi.nlm.nih.gov/sra/?term=SRR21079300) https://www.ncbi.nlm.nih.gov/nuccore/CP102942 https://www.ncbi.nlm.nih.gov/nuccore/CP102943

## Value of the Data

• These data are available for analysis by researchers to understand the molecular epidemiology of *Stenotrophomonas maltophilia*.
• These data will be used to improve surveillance and prediction of hospital outbreaks of *Stenotrophomonas maltophilia*.
• Whole genome sequencing data provide information about genomic determinants and antimicrobial resistance (AMR) genes of *Stenotrophomonas maltophilia* strain SCAID WND1-2022 (370).
• These data should be used by researchers and public health officers for surveillance and monitoring of *Stenotrophomonas maltophilia* to prevent the emergence of highly resistant strains.
• The data can be used by researchers for genomics, proteomics and other evolutionary studies.

## 1 Objective

Antimicrobial resistance (AMR) is the main threats to human health. Monitoring the spread of genetic determinants and evaluating the etiologic and taxonomic composition of nosocomial pathogens will allow the timely identification of threats to human health. Data was obtained during the implementation of the ID #BR09458960 grant to create a collection of multi drug resistant bacterial strains causing nosocomial infections. The isolated strain *Stenotrophomonas maltophilia* SCAID WND1-2022 (370) will be used as a model organism to examine the development of virulence and antibiotic resistance. The data submitted demonstrate the genetic and epigenetic properties of the multidrug-resistant clinical isolate belonging to Gram-negative pathogens. These data can be helpful for the surveillance and prediction of outbreaks of *Stenotrophomonas maltophilia*-related hospital infections.

## 2 Data Description

*Stenotrophomonas maltophilia* is a major intra-hospital pathogen characterized by an extended multidrug resistance. Severe infections caused by *S. maltophilia* are associated with a high mortality rate, especially among people with weakened immunity [1]. Strategies are therefore necessary to improve the diagnostic of the infection and the treatment outcomes.

PacBio Sequel-I (Pacific Biosciences) sequencing platform was used to generate 103,602 single-end reads of an average length 8308 bp N50 – 10,885 bp from a template DNA extracted from cultivated clinical isolate *Stenotrophomonas maltophilia* SCAID WND1-2022 (370). The sequencing statistics is summarized in Table 1. The DNA reads were quality filtered and trimmed prior to assembly followed by genome annotation.

**Table 1:** Genome sequence characteristic of *S. maltophilia* SCAID WND1-2022 (370).

Index	Value
Library type	Single-end
Total length reads	802,012,239
Raw reads generated	103,602
Mean read length	8308 bp
Genome size	4,880,425
GC content	66.2%

*De novo* assembly of the quality-controlled reads produced 2 circular contigs corresponding to one bacterial chromosome and one conjugative plasmid (Table 2).

**Table 2:** Replicons of *S. maltophilia* SCAID WND1-2022 (370) and genome annotation.

Features	Chromosome	Plasmid
GC-content	66.2%	61.8%
CDSs (total)	4433	40
rRNA	13	0
tRNA	74	0
Pseudo Genes (total)	44	0
Methylated AC*C*TC sites	6407 (99.9%)	71 (100%)

**Fig. 1:**
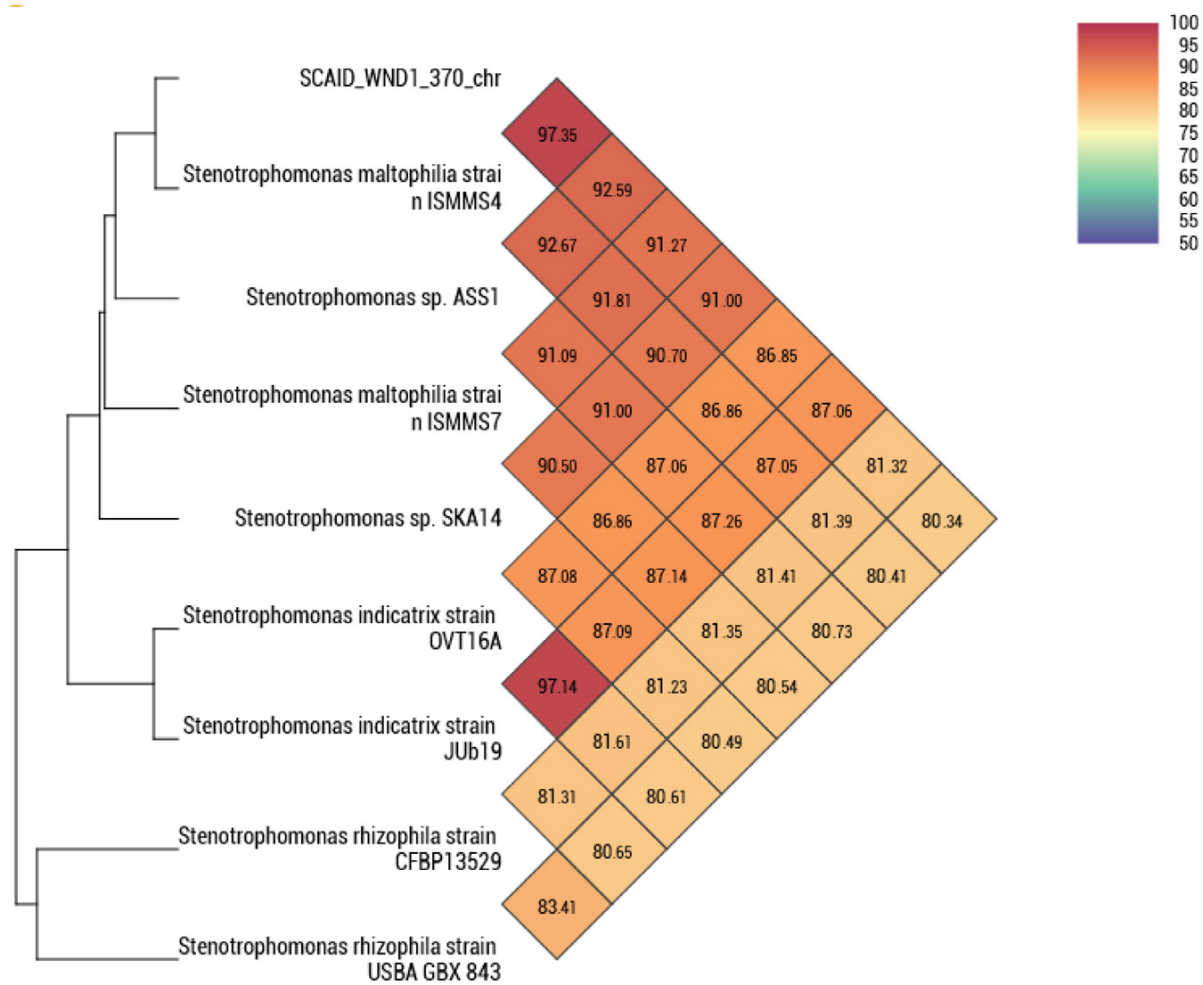
Phylogenetic relations of the strain SCAID WND1-2022 (370) with the selected reference *Stenotrophomonas* strains available from the GenBank database. Calculated pair-wise OrthoANI values are shown in the distance matrix.

Both replicons were methylated at third cytosine residues of AC*C*TC motifs. This methylation is likely associated with a type III restriction-modification methyltransferase found on the chromosome. Taxonomic affiliation of the strain was predicted by whole genome comparison using calculated genome-to-genome distance calculator (GGDC) and OrthoANI values. The most closely related microorganism found in the GenBank database was *Stenotrophomonas maltophilia* ISMMS4. (Table 3 and Fig. 1).

**Table 3:** Taxonomic identification of SCAID WND1-2022 (370).

Strain	Accession	Size (bp)	GC%	OrthoANI value (%)	GGDC distance
*S. maltophilia* ISMMS4	NZ_JZIU01000001.1	4 734 405	66,2	97,35	0,027
*S. maltophilia* ISMMS7	NZ_JZTX01000001.1	4 685 044	66,9	91,27	0,088
*S. indicatrix* OVT16A	NZ_JAHXYT010000001.1	4 166 325	66,2	86,85	0,132
*S. indicatrix* JUb19	NZ_JACAWX010000001.1	4 577 105	66,3	87,06	0,131
*S. rhizophila* CFBP13529	QGAK01000001.1	1 928 423	67,2	81,32	0,175
*S. rhizophila* USBA GBX 843	RCDC01000004.1	2 619 287	66,4	80,34	0,185
*Stenotrophomonas* sp. ASS1	NZ_CP031167.1	4 564 481	66,4	92,59	0,077
*Stenotrophomonas* sp. SKA14	NZ_DS999412.1	5 017 753	66,0	91,00	0,093

**Fig. 2:**
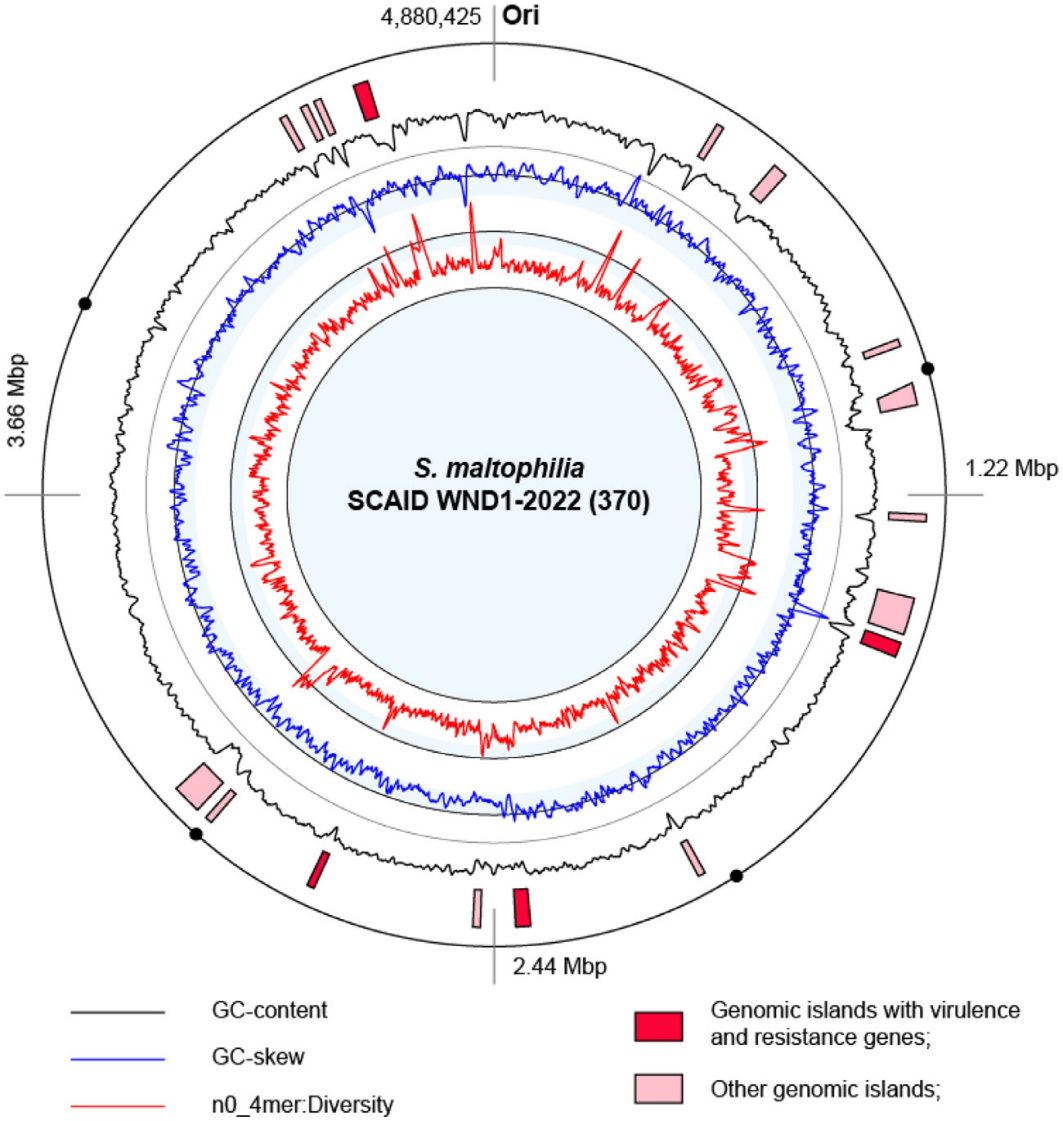
Atlas representation of the chromosome of *S. maltophilia* SCAID WND1-2022 (370). Replication origin is marked as Ori. Colored histograms from the uppermost (black) to the innermost (red) show respectively fluctuations of GC-content, GC-skew and deviations of frequencies of tetranucleotide of 5 kbp sliding windows from the whole genome pattern of tetranucleotides. Pink and red blocks depict locations of identified horizontally acquired genomic islands (GIs). Red GIs comprise virulence and/or drug resistance genes.

Fig. 2 shows an atlas representation of the circular chromosome of *S. maltophilia* SCAID WND1-2022 (370). 17 loci on the chromosome were predicted as putative genomic islands (GIs). Locations of the genomic feature were counted on the atlas clockwise starting from the replication origin (Ori) located ∼400 bp upstream of *dnaA* encoding chromosomal replication initiator protein.

Clinical isolates of *S. maltophilia* are characterized with high-level intrinsic and acquired resistance to a wide range of antibiotics [2]. Drug resistance (DR) and virulence (Vir) genes of the strain *S. maltophilia* SCAID WND1-2022 (370) were predicted using curated public database and gene annotation. The repertoire of DR genes includes 61efflux pumps organized in 26 operons of RND, EmbrAB-OMF, MdtABC-TolC and several other unrecognized types; 14 heavy metal efflux pumps; 14 beta-lactamases including one metallo-beta-lactamase; 3 small multidrug resistance family (SMR) proteins and one multidrug resistance protein NorM (Supplementary material). Vir proteins were identified by a search through the VFDM database. This identification revealed 56 genes involved in motility and biofilm formation; 37 adhesion and pilus proteins; 21 immune modulators; 30 effector transporters; 32 siderophores and iron transportation genes; 8 invasion proteins and toxins; 5 stress response proteins (Supplementary material). Many DR and Vir genes were located in the core part of the chromosome. Several operons of heavy metal resistance genes, multidrug efflux pumps, invasion and immune modulation proteins were found in GIs. These GIs are highlighted in Fig. 2 by red color.

**Fig. 3:**
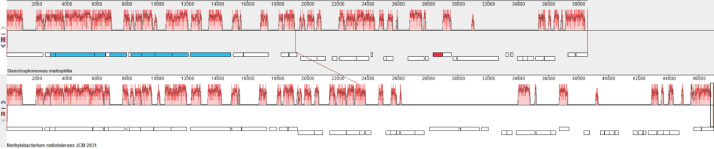
Alignment of sequences of the plasmid found in *S. maltophilia* SCAID WND1-2022 (370) on the top and the plasmid pMRAD02 from *Methylobacterium radiotolerans* JCM 2831 selected as the closest relative found in the NCBI database by a BLASTN search. Histograms show the level of nucleotide sequence similarity between homologous fragments of the plasmids. Boxes below the histograms show gene locations. Genes encoding the T4SS system are shown in blue.

The identified plasmid has another GC-content then the chromosome. A BLASTN search through the NCBI nr/nt database revealed the most similar plasmid pMRAD02 (CP001003) from *Methylobacterium radiotolerans* JCM 2831 and several plasmids from phytopathogenic *Xanthomonas citri*. Alignment of the sequences of the plasmid of *S. maltophilia* SCAID WND1-2022 (370) and pMRAD02 is shown in Fig. 3. An elaborated type IV secretion system (T4SSa) is shared by these two plasmids. No genes associated with antimicrobial resistance were detected on the plasmid.

The isolated strain *S. maltophilia* SCAID WND1-2022 (370) will be used as a model organism to examine the development of virulence and antibiotic resistance by this important pathogen causing acute hospital infections.

*Stenotrophomonas maltophilia* SCAID WND1-2022 (370) was deposited at NCBI GenBank with the accession numbers: CP102942 (https://www.ncbi.nlm.nih.gov/nuccore/CP102942) for the chromosome and CP102943 (https://www.ncbi.nlm.nih.gov/nuccore/CP102943) for the plasmid under the BioProject: PRJNA754843 (https://www.ncbi.nlm.nih.gov/bioproject/?term=PRJNA754843).

This data could be used for a more in-depth analysis of antimicrobial resistance and bacterial virulence factors, as well as to improve surveillance and prediction of hospital outbreaks caused by *S. maltophilia.*

## 3 Experimental Design, Materials and Methods

### 3.1 Sample Collection and Isolation of *Stenotrophomonas maltophilia* Strain SCAID WND1-2022 (370)

The isolate was obtained in 2022 from the intensive care unit at the Syzganov's National Surgery Center in Almaty, Kazakhstan. It has been approved by the Committee of Institutional Animal Care and Use in the Scientific Center for Anti-Infectious Drugs (SCAID), Almaty (ID: #2 from 09.16.2020). The sample was taken on a patient with bacterial septicemia. The sample was swabbed out of a purulent wound during a post-mortal autopsy after a fatal sepsis.

In brief, the sample was cultured on selective and differential media (MacConkey agar) before proceeding with the biochemical testing and Gram staining. Routine confirmation of identity was by NEFERMtest kit (Erba Lachema s.r.o., Czech Republic). A wide spectrum of biochemical tests such as urea hydrolysis, acid from lactose, acid from galactose, arginindihydrolase, acid from mannitol, acid from maltose, ornithindecarboxylase, acid from trehalose, acid from cellobiose, lysindecarboxylase, acid from xylose, acid from saccharose, acetamide utilization, acid from L-arabinose, acid from myo-inositol, β-glukosidase, α-galactosidase, γ-glutamyle-transferase, N-acetyl-glucosaminidase, β-galactosidase (ONPG), phosphatase production, citrate utilization, malonate utilization, aesculine hydrolysis allow identified the isolate with 99.63% of reliability. The ESBL screening and antibiotic sensitivity profiles were measured according to Kirby-Bauer disk diffusion methods as per Clinical and Laboratory Standards Institute (CLSI) guidelines and interpretative criteria [3]. The disk diffusion method was used to determine the antimicrobial resistance to the following antibiotics: oxacillin (1 µg/disk), cefazolin (30 µg/disk), amoxicillin (10 µg/disk), gentamycin (30 µg/disk), levofloxacin (5 µg/disk), amikacin (10 µg/disk), erythromycin (10 µg/disk), meropenem (10 µg/disk), azithromycin (30 µg/disk), ceftriaxone (30 µg/disk), tobramycin (30 µg/disk), carbenicillin (100 µg/disk), ampicillin (10 µg/disk), cefepime (30 µg/disk), clindamycin (10 µg/disk) (Supplementary material).

### 3.2 DNA Isolation, Genome Sequencing, Assembly, and Annotation

For DNA extraction, culture was grown on nutrient agar (Nutrient Agar, HiMedia) for 24 h at 37 °C. DNA was extracted using PureLink Genomic DNA Mini Kit (Invitrogen, USA) following the manufacturer's recommendations. The quality and quantity of the resulting DNA samples were determined using the NanoDrop 2000c spectrophotometer (Thermo Scientific, USA) at the optical wavelengths of 260 and 280 nm.

The DNA library was sequenced using the PacBio Sequel-I (Pacific Biosciences) sequencing platform by Macrogen (Seoul, Korea) as described before [4]. Further processing of the DNA reads was performed using software tools as described below with default parameter settings if not indicated otherwise.

In total, 103,602 single-end reads with an average length of 8308 bp (N50 – 10,885 bp) were obtained. The DNA reads were quality controlled and checked for remaining adapters using LongQC v1.2.0c [5]. Filtering and trimming returned 84,826 reads of the total length 802,012,239 bp. Genome assembly was performed using Canu v2.0 [6]. The original DNA reads were mapped to the scaffolds using pbmm2 (SMRT Link v10.1.0.119588) for error correction. Consensus sequences were generated from the alignments using the gcpp arrow algorithm (SMRT Link v10.1.0.119588) and also using a pipeline of samtools-1.10, bcftools-1.7 and vcfutils.pl utilities. The consensus sequences were annotated using NCBI Prokaryotic Genome Annotation Pipeline (PGAP) [7].

The raw reads were deposited under the BioProject number PRJNA754843 with SRA accession SRR21079300. The complete chromosomal sequence was deposited under the accession number - CP102942 and the number of plasmid sequence is CP102943.

Genome-scale sequences were aligned by the program MAUVE 20150226 [8].

### 3.3 Taxonomic Identification

OrthoANI v.0.93.1 [9] was used to identify strain by estimating genomic distance without alignment and computing mean nucleotide identity.

### 3.4 Screening for Drug Resistance Genes and Virulence Factors

Drug resistance genes were identified by Abricate v.1.0.1 (https://github.com/tseemann/abricate) using CARD (data update 2021-Mar-27) [10] and MEGARES 2.00 (data update 2021-Mar-27) databases [11]. Only the genes with the coverage greater than 90% were included. The search for virulence factors was carried out using the Galaxy platform (https://usegalaxy.org/) via vfdb database [12].

### 3.5 Genomic Islands Identify and DNA Methylation Profiling

Mobile horizontally acquired genomic islands (GIs) were identified using SeqWord Genomic Island Sniffer [13]. Sequence similarity between GIs was estimated by comparison of frequencies of tetranucleotides in their composition [14].

Identification of methylated nucleotides and motifs of DNA methylation was performed using programs ipdSummary and motifMaker of the package SMRT Link v10.1.0.119588 as described previously [15].

## Ethics Statements

The protocol was approved by the Committee of Institutional Animal Care and Use in the Scientific Center for Anti-Infectious Drugs (SCAID), Almaty (ID: #2 from 09.16.2020).

## CRediT authorship contribution statement

**Ilya Korotetskiy:** Conceptualization, Writing – original draft, Writing – review & editing, Project administration. **Ardak Jumagaziyeva:** Methodology, Data curation. **Bahkytzhan Kerimzhanova:** Validation, Writing – review & editing, Supervision. **Oleg Reva:** Software, Visualization, Writing – original draft, Writing – review & editing. **Tatyana Kuznetsova:** Formal analysis, Writing – review & editing. **Sergey Shilov:** Formal analysis, Investigation. **Ludmila Ivanova:** Investigation, Visualization. **Natalya Zubenko:** Visualization, Data curation. **Raikhan Parenova:** Visualization, Formal analysis. **Zhanar Iskakbayeva:** Methodology, Data curation. **Bolatbek Baimakhanov:** Resources, Data curation. **Aimana Bekmuhamedova:** Resources, Data curation.

## Declaration of Competing Interest

The authors declare that they have no known competing financial interests or personal relationships that could have appeared to influence the work reported in this paper.

## Data Availability

Dataset Virulence and drug resistance of Stenotrophomonas maltophilia SCAID WND1-2022 (370) (Original data) (Mendeley Data)

## Appendix Supplementary materials

Image, application 1
